# Detecting aortic valve stenosis based on the non-invasive blood pressure waveform—a proof of concept study

**DOI:** 10.1007/s11357-024-01136-w

**Published:** 2024-03-20

**Authors:** Eline Kho, Jimmy Schenk, Alexander P. J. Vlaar, Marije M. Vis, Marije Wijnberge, Lotte B. Stam, Martijn van Mourik, Harald T. Jorstad, Henning Hermanns, Berend E. Westerhof, Denise P. Veelo, Bjorn J. P. van der Ster

**Affiliations:** 1 Department of Anaesthesiology, Amsterdam UMC, University of Amsterdam, Amsterdam Cardiovascular Sciences, Amsterdam, the Netherlands; 2 Department of Epidemiology and Data Science, Amsterdam UMC, University of Amsterdam, Amsterdam Public Health, Amsterdam, the Netherlands; 3 Department of Intensive Care, Amsterdam UMC, University of Amsterdam, Amsterdam, the Netherlands; 4 Laboratory of Experimental Intensive Care and Anaesthesiology, Amsterdam UMC, University of Amsterdam, Amsterdam, the Netherlands; 5 Department of Cardiology, Amsterdam UMC, University of Amsterdam, Amsterdam Movement Sciences, Amsterdam Cardiovascular Sciences, Amsterdam, the Netherlands; 6 Department of Pulmonary Medicine, Amsterdam UMC, Vrije Universiteit Amsterdam, Amsterdam Cardiovascular Sciences, Amsterdam, Netherlands; 7 Department of Neonatology, Radboud University Medical Center, Radboud Institute for Health Sciences, Amalia Children’s Hospital, Nijmegen, Netherlands

**Keywords:** Blood pressure, Aortic valve stenosis, Non-invasive blood pressure, Nexfin, Prediction model

## Abstract

The incidence of aortic valve stenosis (AoS) increases with age, and once diagnosed, symptomatic severe AoS has a yearly mortality rate of 25%. AoS is diagnosed with transthoracic echocardiography (TTE), however, this gold standard is time consuming and operator and acoustic window dependent. As AoS affects the arterial blood pressure waveform, AoS-specific waveform features might serve as a diagnostic tool. Aim of the present study was to develop a novel, non-invasive, AoS detection model based on blood pressures waveforms. This cross-sectional study included patients with AoS undergoing elective transcatheter or surgical aortic valve replacement. AoS was determined using TTE, and patients with no or mild AoS were labelled as patients without AoS, while patients with moderate or severe AoS were labelled as patients with AoS. Non-invasive blood pressure measurements were performed in awake patients. Ten minutes of consecutive data was collected. Several blood pressure-based features were derived, and the median, interquartile range, variance, and the 1st and 9th decile of the change of these features were calculated. The primary outcome was the development of a machine-learning model for AoS detection, investigating multiple classifiers and training on the area under the receiver-operating curve (AUROC). In total, 101 patients with AoS and 48 patients without AoS were included. Patients with AoS showed an increase in left ventricular ejection time (0.02 s, *p* = 0.001), a delayed maximum upstroke in the systolic phase (0.015 s, *p* < 0.001), and a delayed maximal systolic pressure (0.03 s, *p* < 0.001) compared to patients without AoS. With the logistic regression model, a sensitivity of 0.81, specificity of 0.67, and AUROC of 0.79 were found. The majority of the population without AoS was male (85%), whereas in the population with AoS this was evenly distributed (54% males). Age was significantly (5 years, *p* < 0.001) higher in the population with AoS. In the present study, we developed a novel model able to distinguish no to mild AoS from moderate to severe AoS, based on blood pressure features with high accuracy. Clinical registration number: The study entailing patients with TAVR treatment was registered at ClinicalTrials.gov (NCT03088787, https://clinicaltrials.gov/ct2/show/NCT03088787). The study with elective cardiac surgery patients was registered with the Netherland Trial Register (NL7810, https://trialsearch.who.int/Trial2.aspx?TrialID=NL7810).

## Introduction

Severe aortic stenosis (AoS) is the most common valvular heart disease in Europe and North America [1]. The incidence of AoS increases with age, ranging from 0.2% in patients of 50–59 years to almost 10% in 80–89 years old patients [2]. AoS has a drastic impact on quality of life due to its debilitating symptoms, such as impaired exercise tolerance and decreased functionality, syncope, and other severe exercise-induced complaints [3]. Once diagnosed, if untreated, symptomatic severe AoS has a yearly mortality rate of 25% [4]. Examination of the potential stenosis of the aortic valve often occurs after the arise of symptoms and currently, transthoracic echocardiography (TTE) is the gold standard to confirm diagnosis and assess the severity of AoS. Once severe AoS is symptomatic, early intervention is strongly recommended [5]. The only effective treatment for severe AoS is valve replacement, either by surgical aortic valve replacement (SAVR) or transcatheter aortic valve replacement (TAVR).

Stenosis of the aortic valve diminishes the aortic valve area, increases afterload by adding a valvular resistance, and can result in both progressive hypertrophy of the left ventricle, and a reduced systolic coronary flow velocity, compromising subendocardial perfusion [6–8]. In addition, because of the added resistance, left ventricular ejection time (LVET) increases [9]. It is possible that the treatment of AoS after the manifestation of AoS related symptoms is suboptimal in some patients, as pathophysiological changes could be irreversible [10]. Early diagnosis of an evolving AoS could prove helpful in preventing potentially irreversible changes such as decline in left ventricular function [11]. However, in most cases, TTE is only performed when indicated by patients’ symptoms. Furthermore, TTE is known to be time consuming and operator and acoustic window dependent [12]. A more simple, low cost, non-invasive and feasible way to detect AoS would therefore be of added value.

AoS causes a delayed pressure rise in the aorta, and a prolonged systolic ejection period [13, 14]. As this affects the blood pressure waveform, this change in morphology can potentially be measured more distally in the vascular tree. In patients with AoS, a prolongation of left ventricular ejection and upstroke time, and a less steep slope are expected [15–18]. In this study, we aimed to create a machine-learning derived diagnostic model to detect severe AoS based on non-invasive blood pressure waveform features.

## Methods

Data of two prospective, single-centre studies was combined, comprising a population of patients with and without AoS. The first study included patients with AoS who underwent elective TAVR, recruited from March 2017 until February 2019, registered at ClinicalTrials.gov (NCT03088787). The second study consisted of patients who underwent elective cardiac surgery, including SAVR. These patients were recruited from October 2019 until May 2022, and registered with the Netherland Trial Register (NL7810).

We excluded patients with a body weight below 40 kg, age younger than 65 years, a congenital unicuspid or bicuspid valve, mechanical aortic valve prosthesis, atrial fibrillation or flutter, a mitral valve insufficiency categorized as higher than mild, no TTE available to the procedure, or inability to perform non-invasive blood pressure measurements.

### Transthoracic echocardiography

Severity of AoS was derived from TTE reports extracted from the electronic patient records, executed at least within 6 months before either TAVR or surgery, or within 12 months in case moderate or severe AoS was detected. AoS was graded according to the EAE/ASE guideline [19]. This always included, but was not limited to, assessment of: AoS jet velocity, the trans-aortic gradient and the valve area by continuity equation. The final grading severity was at the discretion of accredited echocardiographers. Patients with mild or no AoS were classified as no AoS patients, and patients with a moderate or severe AoS were considered AoS patients.

### Non-invasive blood pressure monitoring

Non-invasive blood pressure data was obtained in all patients using a finger cuff with built-in light emitting- and receiving plethysmography diodes (^cc^Nexfin, Edwards Lifesciences, Irvine, CA, USA), applied to the middle or index finger. Within the device, the finger blood pressure curve was automatically transformed to the brachial blood pressure waveform with a sample frequency of 200 Hz [20]. Non-invasive measurement of blood pressure has shown to be accurate in patients with severe aortic stenosis [21, 22].

Blood pressure data was collected shortly before procedure until either induction of general anaesthesia (in case of surgery) or local anaesthesia (in case of TAVR) was administered. Two researchers (EK and JS) manually selected a segment of ten minutes of consecutive data. In case of artefacts in the data, a shorter data segment was selected, with a minimum length of 3 min.

### Data analyses

The ^cc^Nexfin automatically calculates several parameters, such as the systolic (SAP), mean (MAP), and diastolic (DAP) arterial blood pressure [20]. Furthermore, the interbeat interval (IBI), heart rate (HR), left ventricular ejection time (LVET), stroke volume (SV), stroke volume index (SVI) cardiac output (CO), cardiac index (CI), systemic vascular resistance (SVR), systemic vascular resistance index (SVRI), and an estimated index of left ventricular contractility (dP/dt, the maximum value of the first time-derivative of pressure), are automatically calculated.

From these derived parameters, several extra features were calculated offline. First, the pulse pressure (PP) was calculated subtracting DAP from SAP; stroke work (SW) was calculated multiplying SV with MAP [23]. The instantaneous baroreflex sensitivity (xBRS), a measure of autonomic function, was computed and expressed as millisecond (ms) change in IBI per mmHg change in SAP [24]. Here, the regression line with the highest correlation between the two changes, while shifting in time, was calculated. The slope of this line was defined as the gain, and the corresponding shift in time was described as the delay [24].

From the raw blood pressure data, several extra features were calculated for individual beats. After applying the smoothing Savitzky-Golay filter, the timing of SAP, dicrotic notch and corresponding time, area under the curve (AUC) of SAP/DAP, based on the AUC of the beat until/from the dicrotic notch, were calculated. The dicrotic notch was calculated by averaging the time of the second maximum of the first and second derivative of the raw blood pressure beat [25]. Furthermore, for each beat, the area under the curve, but above the dicrotic notch, maximum slope of the up- and down-stroke of the systolic part of the beat, based on the maximum and minimum of the first derivative, were calculated.

### Statistical methods

In total, 27 features based on non-invasive blood pressure measurement were derived and used to calculate the final features used for the model. From these features, the median, interquartile range (IQR), variance, the 1st and 9th decile of the change were derived. Next, the features were divided by the patients’ age, to adjust for age-dependent differences, and then split into a training and a test set. The training set was used to derive the most optimal model, whereas the test set was used to test this model. The training set was based on 75% of the data, and consisted of an imbalanced set (75 patient with AoS, and 36 with no AoS). To create a more balanced dataset, the set of 36 patients without AoS was oversampled with the Synthetic Minority Over-sampling Technique (SMOTE), to match the 75 patient with AoS [26]. Next, the training dataset was normalized with MinMaxScaler (Scikit-Learn 1.1.3) and used as the input to several classifiers; logistic regression, K-nearest neighbours, decision tree, support vector machine, and random forest. Additionally, the hyperparameters of all classifiers were optimized through a grid search with four-fold cross validation. Training was performed towards the highest possible area under the receiver operating curve (AUROC).

Difference between patients with and without AoS was tested statistically with the unpaired t-test or Wilcoxon rank sum test in case of non-parametric data, or with the Fisher’s exact test when it concerned discrete data. For descriptive purposes, significant differences of the features between the two populations were calculated based on the value of the features before correcting for age. Descriptive data are presented as mean with (SD) or median with (1st–3rd quartile), when applicable. A *p*-value < 0.05 was considered statistically significant. All data and statistical analyses were performed with Matlab (Version 2020b, the Mathworks Inc., Nattick, MA, USA) or Python (Version 3.9, package: Scikit-learn 1.1.3).

## Results

**Fig. 1:**
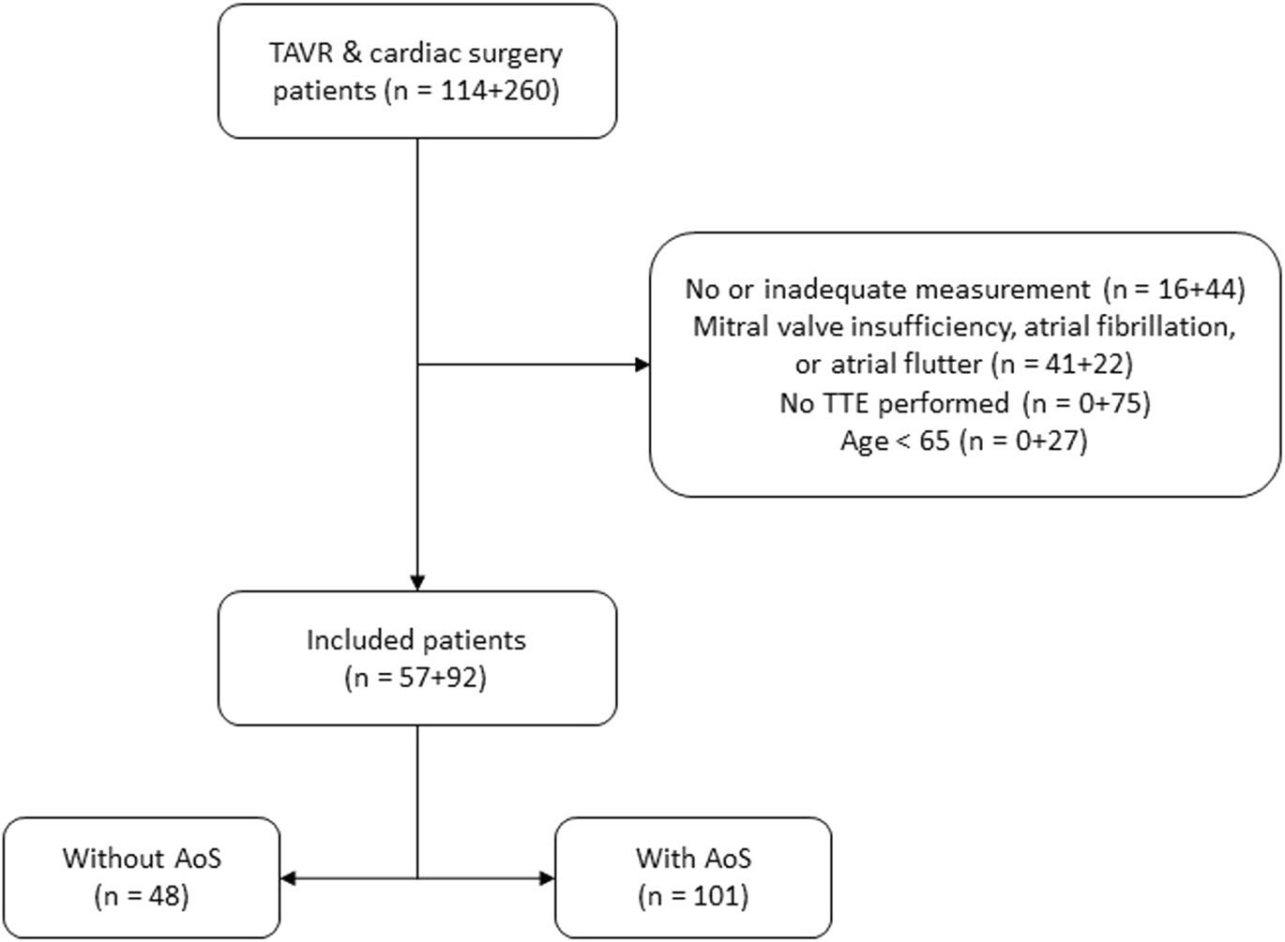
Study flow diagram

In the TAVR sample, 114 patients were included, of whom 57 were eligible for further analyses (Fig. 1). In the cardiac surgery sample, 260 patients were included, of whom 92 were eligible for further analyses.

In the combined sample, 101 (68%) were classified as AoS patients and 48 (32%) as patients with no AoS. Study population characteristics of the patients can be found in Table 1. The majority of the population without AoS was male (41 vs 7, *p* < 0.001), while the population with AoS was evenly distributed (46 females vs 55 males, *p* = 0.260). Compared to patients without AoS, patients with AoS were older (78 (73–83) vs 73 (68–77) years), had a lower bodyweight (76 (70–87) vs 83 (75–92) kg), and had a lower length (170 (164–178) vs 176 (172–179) cm). For both populations, the majority had an American Society of Anesthesiologists (ASA) classification of III (80% vs 83%). The median (1st to 3rd quartile) duration of the selected blood pressure data for patient with AoS was 600 (435–600) seconds, whereas for patients without AoS this was 400 (250–600) seconds, a significant difference of 200 s (95% CI: 114–283), *p* < 0.001. In the population with AoS, 9 out of 101 patients were classified as having low flow low gradient.

**Table 1:** Patient characteristics

	*No AoS (n:48)*	*AoS (n:101)*	*p-value*
*Female*	7 (15%)	46 (46%)	< 0.001^a^
*Age, years*	73 (68–77)	78 (73–83)	< 0.001^**b**^
*Weight, kg*	83 (75–92)	76 (70–87)	0.009^b^
*Height, cm*	176 (172–179)	170 (164–178)	0.0048^b^
*BMI, kgm*^*−2*^	27.4 (24.7–29.6)	26.4 (24.3–29.3)	0.405^b^
*ASA*			
*I*	0 (0%)	1 (1%)	1.000^a^
*II*	5 (10%)	9 (9%)	0.770^a^
*III*	40 (83%)	81 (80%)	0.823^a^
*IV*	3 (6%)	10 (10%)	0.550^a^
*History of*			
*Myocardial Infarction*	10 (21%)	17 (17%)	0.650^a^
*COPD*	2 (4%)	12 (12%)	0.228^a^
*PAOD*	5 (10%)	11 (11%)	1.000^a^
*Diabetes Mellitus*	10 (21%)	26 (26%)	0.547^a^
*Cerebral Vascular Accident*	7 (15%)	18 (18%)	0.815^a^
*Hypertension*	28 (58%)	68 (67%)	0.360^a^
*NYHA Classification* > *2*	1 (2%)	11 (11%)	0.104^a^
*Aortic Stenosis Grade*			
*None*	39 (81%)	0 (0%)	< 0.001^a^
*Mild*	9 (19%)	0 (0%)	< 0.001^a^
*Moderate*	0 (0%)	14 (14%)	< 0.001^a^
*Severe*	0 (0%)	87 (86%)	< 0.001^a^
*Aortic Valve Area, cm*^*2*^	-	0.82 (0.72–0.94)	-
*Aortic Valve Max Gradient, mmHg*	-	63 (53–80)	-
*Aortic Valve Mean Gradient, mmHg*	-	37 (30–46)	-

Data are presented as median (1st–3rd quartile), or as amount (percentage). ASA: American Society of Anesthesiologists physical status classification system, COPD: Chronic Obstructive Pulmonary Disease, PAOD: Peripheral Arterial Occlusive Disease, NYHA; New York Heart Association

^a^Fisher’s exact test

^b^Wilcoxon rank sum test

When compared to patients without AoS, patients with AoS showed significantly higher values for LVET, AUC SAP, and AUC dicrotic notch, whereas lower dP/dt, SW, AUC DAP, timing of the maximal up- and downstroke were found. Exact differences, confidence intervals and *p*-values can be found in Table 2. Here, the average values of the features before adjusting for age are displayed, while the values after adjusting for age are implemented in the machine-learning model.

**Table 2:** Features before adjusting for age

		*No AoS (n:48)*	*AoS (n:101)*	*Difference* *(95% CI)*	*p-value*
*Median value*	*IBI (s)*	0.88 (0.84–0.95)	0.87 (0.77–0.97)	- 0.01 (-0.07 to 0.05)	0.661
*HR (beats·min*^*−1*^*)*		69 (64–72)	69 (62–78)	0.5 (-4.3 to 5.3)	0.707
*LVET (s)*		32.5 (31.0–34.5)·10^–2^	34.5 (32.5–36.5)·10^–2^	2.0 (0.7 to 3.3)·10^–2^	0.001
*SAP (mmHg)*		155 (135–174)	158 (140–174)	3 (-9 to 15)	0.605
*DAP (mmHg)*		76 (72–87)	75 (68–81)	-1 (-6 to 4)	0.154
*MAP (mmHg)*		105 (94–121)	105 (97–115)	1 (-7 to 8)	0.901
*SV (mL)*		77 (68–91)	71 (61–85)	-6 (-14 to 3)	0.021
*CO (L·min*^*−1*^*)*		5.3 (4.6–6.2)	4.7 (4.1–5.8)	-0.6 (-1.1 to 0.03)	0.062
*SVR (mL·min*^*−1*^*)*		1522 (1346–1859)	1683 (1472–2088)	161 (-40 to 361)	0.076
*SVI*		40 (36–42)	38 (33–43)	-2 (-4 to 1)	0.071
*CI (L·min*^*−1*^*·m*^*2*^*)*		2.7 (2.4–3)	2.6 (2.1–3)	-0.1 (-0.4 to 0.2)	0.310
*SVRI (mL·m*^*2*^*)*		3153 (2755–3500)	3203 (2876–3878)	50 (-269 to 369)	0.322
*dP/dt (mmHg·s*^*−1*^*)*		1185 (881–1509)	858 (636–1163)	-327 (-520 to -134)	< 0.001
*PP (mmHg)*		80 (65–88)	84 (67–94)	4 (-5 to 13)	0.150
*SW (mL·mmHg)*		8650 (6815–10028)	7200 (6003–9328)	-1450 (-2580 to-320)	0.017
*SAP time (s)*		0.17 (0.15–0.19)	0.20 (0.18–0.23)	0.03 (0.015 to 0.045)	< 0.001
*SAP AUC (%)*		45.2 (40.5–49.7)	50.3 (45.8–54.2)	0.1 (0.0 to 0.1)	< 0.001
*DAP AUC (%)*		54.8 (50.3–59.5)	49.8 (45.8–54.2)	-0.1 (-0.1 to -0.0)	< 0.001
*Dicrotic notch time (s)*		0.33 (0.30–0.35)	0.36 (0.34–0.38)	0.04 (0.02 to 0.05)	< 0.001
*Dicrotic notch (mmHg)*		115 (102–132)	114 (101–125)	-1 (-9 to 8)	0.353
*Dicrotic notch AUC (%)*		38.6 (34.2–42.9)	41.7 (37.7–46.4)	0.0 (0.0 to 0.1)	0.002
*Max upstroke (mmHg)*		110 (97–120)	110 (100–120)	1 (-7 to 8)	0.849
*Max upstroke time (s)*		6.0 (5.3–6.5)·10^–2^	7.5 (6.5–9.1)·10^–2^	1.5 (0.7 to 2.2)·10^–2^	< 0.001
*Max downstroke (mmHg)*		130 (117–148)	134 (122–146)	4 (-5 to 13)	0.481
*Max downstroke time (s)*		28.5 (27.0–30.6)·10^–2^	30.5 (28.9–32.5)·10^–2^	2.0 (0.8 to 3.3)·10^–2^	< 0.001
*xBRS gain*		5.1 (3.4–8.0)	4.7 (3.0–7.3)	-0.5 (-2.0 to 1.0)	0.509
*xBRS tau (s)*		3.0 (3.0–3.8)	3.0 (2.9–3.0)	0.0 (-1.2 to 1.2)	0.240

For all features the median (1st–3rd quartile) are represented, before adjusting for age, for descriptive purposes

IBI; InterBeat Interval, HR; Heart Rate, LVET; Left Ventricular Ejection Time, SAP; Systolic Arterial blood Pressure, DAP; Diastolic Arterial blood Pressure, MAP; Mean Arterial blood Pressure, SV; Stroke Volume, CO; Cardiac Output, SVR; Systemic Vascular Resistance, SVI; Stroke Volume Index, CI; Cardiac Index, SVRI; Systemic Vascular Resistance Index, dP/dt; index of left ventricular contractility, PP; Pulse Pressure, SW; Stroke Work, AUC; Area Under the Curve, xBRS; BaroReflex Sensitivity

### Machine learning derived detection model

**Fig. 2:**
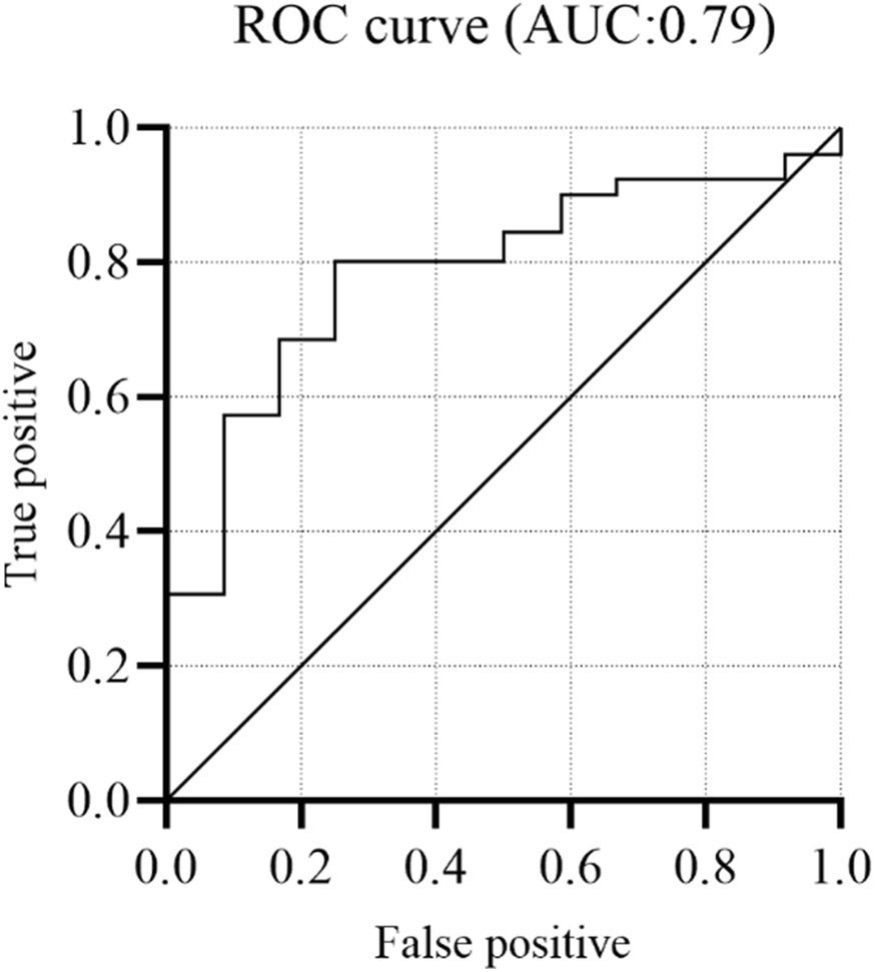
Performance of the logistic regression classifier for detecting aortic valve stenosis. The area under the curve (AUC) indicates the area under the receiver operating characteristic curve

Based on the training set, the best performing (AUROC of 0.93 (SD:0.03)) classifier was logistic regression (parameters listed in Table 3). Applying the model to the test set, an AUROC of 0.79 was found (Fig. 2) with a sensitivity of 0.81 (81% of the patients with AoS are correctly labelled) and a specificity of 0.67 (67% of the patients with no AoS are correctly labelled). The accuracy of the model was 0.76, representing how often the model labels patients correctly (both AoS and no AoS). The positive predictive value was 0.84, so 84% of the patients labelled as AoS actually had AoS, whereas 62% of the patients labelled as no AoS actually had no AoS (negative predictive value: 0.62). In total 8/12 of the cases without AoS were detected by the model, whereas 21/26 of the cases with AoS were detected (Table 4).

**Table 3:** Optimized parameters of the final classifying model

*Parameter*			*Value*
*Tuned parameters*	*Classifier*		Logistic regression
	*C*		10
	*Maximum iteration*		500
	*Fit intercept*		True
	*Penalty*		L2
*Best features, in arbitrary order (n:40)*		*Median*	IBI, HR, SAP, DAP, MAP, SV, CO, SVI, CI, dP/dt, SW, time SAP, time dicrotic notch, dicrotic notch, AUC SAP, AUC DAP, time maximum upstroke, maximum upstroke, maximum downstroke, xBRS tau
	*IQR*		MAP, dP/dt, SW, time dicrotic notch, time maximum upstroke, maximum upstroke
	*Variance*		SVR, SVRI, dP/dt, PP, time maximum downstroke, maximum upstroke
	*1st decile*		SAP, dP/dt, dicrotic notch, maximum upstroke, maximum downstroke
	*9th decile*		SAP, dP/dt, dicrotic notch, maximum upstroke

IBI; InterBeat Interval, HR: Heart Rate, SAP/DAP/MAP; Systolic/Diastolic/Mean Arterial blood Pressure, SV: Stroke Volume, CO: Cardiac Output, SVI: Stroke Volume Index, dP/dt; index of left ventricular contractility, SW: Stroke Work, AUC; Area Under the Curve, xBRS: BaroReflex Sensitivity

**Table 4:** Confusion matrix of the test dataset

		*Predicted model output*		
		No AoS	AoS	Total
*TTE*	No AoS	8	4	12
	AoS	5	21	26
	Total	13	25	38

AoS: Aortic Stenosis, TTE: transthoracic echocardiography

## Discussion

In this study, a detection model based on non-invasive blood pressure waveforms was developed, and showed good to excellent performance in differentiating between no or mild AoS and moderate to severe AoS. The hemodynamic features implemented into the model were to some extent able to differentiate between patients with and without AoS. As the stenosis of the aortic valve results in a diminished opening of the valve, the systolic phase is prolonged: the timing of (maximal) SAP and maximum upstroke occurred later in patients with AoS. This is translated to the increased LVET, and the increased AUC of SAP and dicrotic notch. As heart rate did not differ between the populations, AoS resulted in a shorter diastolic period of the beat. In addition, SV was decreased in patients with AoS.

AoS can be detected through a variety of methods, including physical examination, imaging tests, and cardiac function tests [27–29]. TTE is the gold standard, but expensive, time-consuming and operator and acoustic window dependent [12]. Auscultation showed a sensitivity of 43% and a specificity of 69% for diagnosing significant heart valve disease [27]. Chest radiography and electrocardiography can identify secondary effects of AoS, like left ventricular hypertrophy, which is usually developed after sustained AoS, making early AoS detection more difficult [28]. A deep learning algorithm, based on electrocardiography (ECG), showed a AUROC of 0.88 for detecting significant AoS [29]. This deep learning algorithm is very complex as compared to the more straightforward logistic regression of our model. Besides, only 4% of the patients in the ECG model were diagnosed with AoS, affecting positive and negative predicted values. Comparing both models, our model showed slightly better performance in distinguishing patients, with an accuracy of 0.76 compared to 0.72 of the ECG model. While this ECG model was based on more than 45,000 ECG signals, the novel detection model constructed in this study was based on the data of only 150 patients. As a result, it is likely that model performance could be further optimized in external validation studies that should be performed in the future.

When interpreting model performance, it is important to consider its main goal in clinical practice. High sensitivity represents the ability to correctly identify AoS patients, but does not reckon with false positives. With high specificity, most patients without AoS will be correctly classified, but some patients with AoS could be classified as not having a severe AoS. The goal of this study was to accurately distinguish patients with AoS from patients without AoS, which is best described by the AUROC. The AUROC of the developed detection model was excellent in the training set and very good in the test set, outperformed auscultation [23], and yielded comparable results to an ECG-based detection model [25]. A recent study employing bioinformatics and machine learning identified a novel biomarker of Aortic Valve Calcification. The identified biomarker (fibronectin 1) showed an excellent predictive performance [30]. Both biomarkers and blood pressure waveform derived diagnostic models might prove to be of even further added value if besides detecting severe cases, they would allow classification of AoS severity. This might result in earlier treatment, when progression is fast, diminishing the reduction of quality of life for these patients. In this case, a higher sensitivity could be of interest, especially when the progress of the disease is being monitored.

### Limitations

The constructed model was solely based on non-invasive BP waveform-based features. In our study population, gender showed poor distribution in the population without AoS. This was expected, since patients without AoS were found in the cardiac surgery sample, and the majority of cardiac surgery patients are male, whereas the majority of TAVR patients are female. Consequently, this resulted in a higher weight and height in the population without AoS. In a sample with a more comparable distribution of age and gender, patient characteristics might prove to be beneficial to enhance model performance. To assess its face validity and performance, future studies should further assess potential performance differences based on sex, weight and varying amounts of arterial stiffness. Analysing and adding these features was beyond the scope of this study. Concerning blood pressures, there were no significant differences in SAP, MAP or DAP values between the patients with or without AoS, or comparing TAVR with SAVR. However, some other differences between the two populations were found:

First, there was a difference in number of data between the two populations. Due to the general anaesthesia patients received, there was less time to measure blood pressures compared to patients who did not receive general anaesthesia. The 1st–3rd quartile range for smaller datasets is often broader, however as the smallest dataset was 180 s and most features were calculated every beat, this would not have had major impact in developing our model. The only features based on multiple beats were xBRS gain and tau. Here, 10 s of consecutive data were implemented, which was still considered small enough to not be affected by the available length of data.

Second, there was a difference in age between the two populations. Patients unfit for SAVR, or at high operative risk, received TAVR treatment. This resulted in a higher average age in the TAVR population, and consequently in the patients with AoS. This age difference is a common problem in AoS detection studies [29, 31]. Ageing effects the blood pressure, resulting in an increase in SAP, MAP, and PP [32]. Other age-related hemodynamic alterations are an increase in aortic stiffness and a decrease in the cross section of the peripheral vascular bed. This results in an increase in pulse wave velocity and wave reflection [33, 34]. Comparing this to our study, SAP, MAP, DAP, and PP were not significantly higher in the population with AoS compared to the population without AoS, suggesting that the effect of age was limited.

Third, an unbalanced training set will cause learning algorithms to be biased towards the majority class. Therefore, oversampling was applied with SMOTE. With oversampling, synthetic data is generated based on the actual data instead of copying existing data, and no information is lost. Disadvantage of oversampling is that noise can be introduced in the data, resulting in decrease of model performance. However, a good performance was still found with the model, where a ROC-AUC of 0.79 represents good performance.

A last limitation of this study was the decision to restrict our sample to patients with isolated AoS, i.e. without other heart (valve) diseases. The goal of this study was to assess whether patients with AoS could be distinguished from those without AoS. However, as a consequence, generalizability of this model is limited to this specific population, and has not yet been externally validated in this population. In future studies, we plan to analyse and optimize model performance in a more heterogeneous sample of the population, by incorporating patients with other/mixed heart (valve) diseases. Furthermore, we plan to assess model performance in patients suspected of low flow, low gradient AoS, as the model might provide insight in the necessity to perform additional (burdensome) examination such as a stress test and CT-scan.

## Conclusion

A machine-learning model using non-invasive finger arterial waveform analysis is able to detect moderate and severe aortic stenosis with high sensitivity and adequate specificity. External, independent validation of our model should be performed to assess whether this non-invasive, easy-to-use model may be implemented in clinical practice to detect severe aortic stenosis.

## Data Availability

The data supporting the results of this study are available from the corresponding author upon any reasonable request.

## Abbreviations

AoS: Aortic stenosis
AUC: Area under the curve
AUMC: Amsterdam University Medical Centers
AUROC: Area under the receiver operating curve
CI: Cardiac index
CO: Cardiac output
COPD: Chronic obstructive pulmonary disease
CVA: Cerebral vascular accident
DAP: Diastolic arterial blood pressure
DM: Diabetes mellitus
dP/dt: Ratio of intraventricular pressure change (index of left ventricular contractility)
HR: Heart rate
IBI: Interbeat interval
LVET: Left ventricular ejection time
MAP: Mean arterial blood pressure
NYHA: New York heart association
PAOD: Peripheral arterial occlusive disease
PP: Pulse pressure
SMOTE: Synthetic minority over-sampling technique
SAP: Systolic arterial blood pressure
SAVR: Surgical aortic valve replacement
SV: Stroke volume
SVI: Stroke volume index
SVR: Systemic vascular resistance
SVRI: Systemic vascular resistance index
SW: Stroke work
TAVR: Transcatheter aortic valve replacement
TTE: Transthoracic echocardiography
xBRS: Instantaneous baroreflex sensitivity

## References

[1] Lindman BR, Clavel M, Mathieu P, Iung B, Otto CM, Pibarot P. Calcific aortic stenosis. Nat Rev Dis Prim. 2016;2(16006). 10.1038/nrdp.2016.6.Calcific 10.1038/nrdp.2016.6 PMC5127286 27188578

[2] Eveborn GW, Schirmer H, Heggelund G, Lunde P, Rasmussen K. The evolving epidemiology of valvular aortic stenosis. the Tromsø Study. Heart. 2013;99(6):396–400. 10.1136/heartjnl-2012-302265. 22942293 10.1136/heartjnl-2012-302265

[3] Dweck MR, Boon NA, Newby DE. Calcific aortic stenosis: A disease of the valve and the myocardium. J Am Coll Cardiol. 2012;60(19):1854–63. 10.1016/j.jacc.2012.02.093. 23062541 10.1016/j.jacc.2012.02.093

[4] Yadgir S, Johnson CO, Aboyans V, et al. Global, Regional, and National Burden of Calcific Aortic Valve and Degenerative Mitral Valve Diseases, 1990–2017. Circulation. 2020;141:1670–80. 10.1161/CIRCULATIONAHA.119.043391. 32223336 10.1161/CIRCULATIONAHA.119.043391

[5] Vahanian A, Beyersdorf F, Praz F, et al. 2021 ESC/EACTS Guidelines for the management of valvular heart disease: Developed by the Task Force for the management of valvular heart disease of the European Society of Cardiology (ESC) and the European Association for Cardio-Thoracic Surgery (EACTS). Rev Esp Cardiol (Engl Ed). 2022;75(6):524. 10.1016/j.rec.2022.05.006. 35636831 10.1016/j.rec.2022.05.006

[6] Kupari M, Turto H, Lommi J. Left ventricular hypertrophy in aortic valve stenosis: Preventive or promotive of systolic dysfunction and heart failure? Eur Heart J. 2005;26(17):1790–6. 10.1093/eurheartj/ehi290. 15860517 10.1093/eurheartj/ehi290

[7] Badeer HS. Biological significance of cardiac hypertrophy. Am J Cardiol. 1964;14(2):133–8. 10.1016/0002-9149(64)90123-7. 14204755 10.1016/0002-9149(64)90123-7

[8] Rajappan K, Rimoldi OE, Dutka DP, et al. Mechanisms of coronary microcirculatory dysfunction in patients with aortic stenosis and angiographically normal coronary arteries. Circulation. 2002;105(4):470–6. 10.1161/hc0402.102931. 11815430 10.1161/hc0402.102931

[9] Benchimol A, Dimond EG, Shen Y. Ejection time in aortic stenosis and mitral stenosis. Am J Cardiol. 1960;5(728). 10.1016/0002-9149(60)90049-7 13798725

[10] Schenk J, Kho E, Rellum S, et al. Immediate reduction in left ventricular ejection time following TAVI is associated with improved quality of life. Front Cardiovasc Med. 2022;9. 10.3389/fcvm.2022.988840 10.3389/fcvm.2022.988840 PMC9523106 36187009

[11] Otto CM, Nishimura RA, Bonow RO, et al. 2020 ACC/AHA Guideline for the Management of Patients With Valvular Heart Disease: A Report of the American College of Cardiology/American Heart Association Joint Committee on Clinical Practice Guidelines. J Am Coll Cardiol. 2021;77(4):e25–197. 10.1016/j.jacc.2020.11.018. 33342586 10.1016/j.jacc.2020.11.018

[12] Mǎrgulescu AD. Assessment of aortic valve disease - a clinician oriented review. World J Cardiol. 2017;9(6):481. 10.4330/wjc.v9.i6.481. 28706584 10.4330/wjc.v9.i6.481 PMC5491466

[13] Sabbah HN, Blick EF, Anbe DT, Stein PD. Effect of turbulent blood flow on systolic pressure contour in the ventricles and great vessels: Significance related to anacrotic and bisferious pulses. Am J Cardiol. 1980;45(6):1139–47. 10.1016/0002-9149(80)90471-3. 7189639 10.1016/0002-9149(80)90471-3

[14] Eleid MF, Nishimura RA. Aortic stenosis and the pulse contour: A true marker of severity? Catheter Cardiovasc Interv. 2020;95(6):1235–9. 10.1002/ccd.28674. 31868287 10.1002/ccd.28674

[15] Matthews MB, Medd WE, Gorlin R. Aortic stenosis: A clinical study. Br Med J. 1955;2(4942):759–63. 10.1136/bmj.2.4942.759. 13250210 10.1136/bmj.2.4942.759 PMC1980935

[16] Hancock EW, Abelmann WH. A clinical study of the brachial arterial pulse form, with special reference to the diagnosis of aortic valvular disease. Circulation. 1957;16(4):572–81. 10.1161/01.CIR.16.4.572. 13461265 10.1161/01.cir.16.4.572

[17] Raber G, Goldberg H. Left ventricular, central aortic, and peripheral pressure pulses in aortic stenosis*. Am J Cardiol. 1958;1(5):572–8. 10.1016/0002-9149(58)90139-5. 13520624 10.1016/0002-9149(58)90139-5

[18] Wood P. Clinical Studies Aortic Stenosis. Am J Cardiol. Published online 1958;553–571. 10.1016/0002-9149(58)90138-3 13520623

[19] Baumgartner H, Hung J, Bermejo J, et al. Recommendations on the echocardiographic assessment of aortic valve stenosis: A focused update from the European Association of Cardiovascular Imaging and the American Society of Echocardiography. Eur Heart J Cardiovasc Imaging. 2017;18(3):254–75. 10.1093/ehjci/jew335. 28363204 10.1093/ehjci/jew335

[20] Bos WJW, Van Goudoever J, Van Montfrans GA, Van den Meiracker AH, Wesseling KH. Reconstruction of brachial artery pressure from noninvasive finger pressure measurements. Circulation. 1996;94(8):1870–5. 10.1161/01.CIR.94.8.1870. 8873662 10.1161/01.cir.94.8.1870

[21] Sokolski M, Rydlewska A, Krakowiak B, et al. Comparison of invasive and non-invasive measurements of haemodynamic parameters in patients with advanced heart failure. J Cardiovasc Med. 2011;12(11):773–8. 10.2459/JCM.0b013e32834cfebb. 10.2459/JCM.0b013e32834cfebb 21941196

[22] Lu SY, Dalia AA. Continuous noninvasive arterial pressure monitoring for transcatheter aortic valve replacement. J Cardiothorac Vasc Anesth. 2021;35(7):2026–33. 10.1053/j.jvca.2021.01.012. 33549488 10.1053/j.jvca.2021.01.012

[23] Schramm W. The units of measurement of the ventricular stroke work: A review study. J Clin Monit Comput. 2010;24(3):213–7. 10.1007/s10877-010-9234-4. 20473780 10.1007/s10877-010-9234-4

[24] Westerhof BE, Gisolf J, Stok WJ, Wesseling AKH, Karemaker JM. Time-domain cross-correlation baroreflex sensitivity: Performance on the EUROBAVAR data set. J Hypertens. 2004;22(7):1371–80. 10.1097/01.hjh.0000125439.28861.ed. 15201554 10.1097/01.hjh.0000125439.28861.ed

[25] Singh O, Sunkaria RK. Detection of onset, systolic peak and dicrotic notch in arterial blood pressure pulses. Meas Control. 2017;50(7–8):170–6. 10.1177/0020294017729958.

[26] Chawla NV, Bowyer KW, Hall LO, Kegelmeyer WP. SMOTE: Synthetic Minority Over-sampling Technique Nitesh. J Artif Intell Res. 2002;16:321–57.

[27] Myerson S, Prendergast B, Gardezi S, Prothero A, Kennedy A, Wilson J. 136 Gp auscultation for diagnosing valvular heart disease. Heart. 2017;103(Suppl 5):A101–2. 10.1136/heartjnl-2017-311726.135.

[28] Maganti K, Rigolin VH, Sarano ME, Bonow RO. Valvular heart disease: Diagnosis and management. Mayo Clin Proc. 2010;85(5):483–500. 10.4065/mcp.2009.0706. 20435842 10.4065/mcp.2009.0706 PMC2861980

[29] Kwon JM, Lee SY, Jeon KH, et al. Deep learning–based algorithm for detecting aortic stenosis using electrocardiography. J Am Heart Assoc. 2020;9(7). 10.1161/JAHA.119.014717 10.1161/JAHA.119.014717 PMC7428650 32200712

[30] Xiong T, Han S, Pu L, et al. Bioinformatics and machine learning methods to identify FN1 as a novel biomarker of aortic valve calcification. Front Cardiovasc Med. 2022;9(February):1–19. 10.3389/fcvm.2022.832591. 10.3389/fcvm.2022.832591 PMC8918776 35295271

[31] Shokouhmand A, Aranoff ND, Driggin E, Green P, Tavassolian N. Efficient detection of aortic stenosis using morphological characteristics of cardiomechanical signals and heart rate variability parameters. Sci Rep. 2021;11(1):1–14. 10.1038/s41598-021-03441-2. 34893693 10.1038/s41598-021-03441-2 PMC8664843

[32] Franklin SS, Gustin W IV, Wong ND, et al. Hemodynamic patterns of age-related changes in blood pressure: The Framingham heart study. Circulation. 1997;96(1):308–15. 10.1161/01.CIR.96.1.308. 9236450 10.1161/01.cir.96.1.308

[33] Casey DP, Nichols WW, Braith RW. Impact of aging on central pressure wave reflection characteristics during exercise. Am J Hypertens. 2008;21(4):419–24. 10.1038/ajh.2007.74. 18246057 10.1038/ajh.2007.74

[34] Nichols WW, O’Rourke MF, Avolio AP, et al. Effects of age on ventricular-vascular coupling. Am J Cardiol. 1985;55(9):1179–84. 10.1016/0002-9149(85)90659-9. 3984897 10.1016/0002-9149(85)90659-9

